# Environmental Factors Modulate Saxitoxins (STXs) Production in Toxic Dinoflagellate *Alexandrium*: An Updated Review of STXs and Synthesis Gene Aspects

**DOI:** 10.3390/toxins16050210

**Published:** 2024-04-30

**Authors:** Quynh Thi Nhu Bui, Biswajita Pradhan, Han-Sol Kim, Jang-Seu Ki

**Affiliations:** 1 Department of Life Science, Sangmyung University, Seoul 03016, Republic of Korea; quynhbui202995@gmail.com (Q.T.N.B.); biohansol0109@gmail.com (H.-S.K.); 2 Department of Biotechnology, Sangmyung University, Seoul 03016, Republic of Korea; pradhan.biswajita2014@gmail.com; 3 Department of Botany, Model Degree College, Rayagada 765017, Odisha, India

**Keywords:** *Alexandrium*, saxitoxin, temperature, salinity, nutrients, light intensity, STX synthesis gene (*sxt*)

## Abstract

The marine dinoflagellate *Alexandrium* is known to form harmful algal blooms (HABs) and produces saxitoxin (STX) and its derivatives (STXs) that cause paralytic shellfish poisoning (PSP) in humans. Cell growth and cellular metabolism are affected by environmental conditions, including nutrients, temperature, light, and the salinity of aquatic systems. Abiotic factors not only engage in photosynthesis, but also modulate the production of toxic secondary metabolites, such as STXs, in dinoflagellates. STXs production is influenced by a variety of abiotic factors; however, the relationship between the regulation of these abiotic variables and STXs accumulation seems not to be consistent, and sometimes it is controversial. Few studies have suggested that abiotic factors may influence toxicity and STXs-biosynthesis gene (*sxt*) regulation in toxic *Alexandrium*, particularly in *A. catenella*, *A. minutum*, and *A. pacificum*. Hence, in this review, we focused on STXs production in toxic *Alexandrium* with respect to the major abiotic factors, such as temperature, salinity, nutrients, and light intensity. This review informs future research on more *sxt* genes involved in STXs production in relation to the abiotic factors in toxic dinoflagellates.

## 1. Introduction

Photosynthetic organisms occur in vast quantities in aquatic environments, and they are mostly known as phytoplankton. Not just in freshwater environments, but also in marine ecosystems, phytoplankton play an important role as primary producers [1,2,3,4,5,6]. The organisms are very diverse, varying from photosynthesizing bacteria to plant-like eukaryotic algae. They are classified by pigments and important groups that include diatoms, dinoflagellates, and green algae, although many other groups are represented [7].

**Figure 1:**
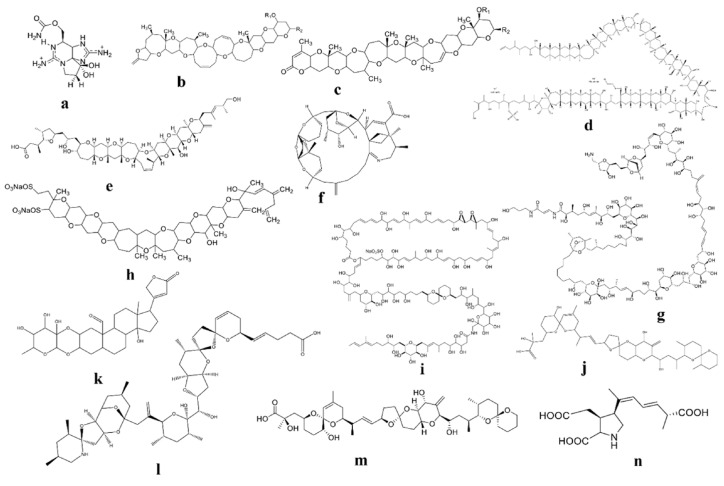
Molecular structures of different hazardous toxins derived from phytoplankton: (**a**) saxitoxins (STXs); (**b**) brevetoxin type A; (**c**) brevetoxin type B; (**d**) maitotoxins (MTXs); (**e**) ciguatoxins (CTXs); (**f**) pinnatoxins (PnTXs); (**g**) palytoxins (PLTXs); (**h**) yessotoxins; (**i**) zooxanthellatoxins; (**j**) dinophysistoxins; (**k**) karlotoxins; (**l**) azaspiracids (AZAs); (**m**) okadaic acid; and (**n**) domoic acid.

Dinoflagellates are a wide category of unicellular microeukaryotes, and half of them are photosynthetic [8]. Thus, they play a crucial role in primary production and coral reef formation in aquatic environments [1,8,9]. They could be a new source of bioactive secondary metabolites, including toxins such as amphidinolides, amphidinol, azaspiracid, ceramide, spirolides, and symbioramide [10,11]. Some dinoflagellates, however, are the main cause of harmful algal blooms (HABs) and produce poisonous substances [12]. The most damaging dinoflagellate toxins are saxitoxin (STX) and its derivatives (STXs), brevetoxins (BTXs), maitotoxins (MTXs), ciguatoxins (CTXs), pinnatoxins (PnTXs), palytoxins (PLTXs), yessotoxins, zooxanthellatoxins, dinophysistoxins, karlotoxins, azaspiracids (AZAs), okadaic acid, and domoic acid [12]. The molecular structures of their representative toxins are displayed in Figure 1.

Among those toxic compounds, STXs, also known as paralytic shellfish toxins (PSTs), are neurotoxic alkaloids naturally produced by certain marine dinoflagellates [13]. PSTs block voltage-gated sodium channels of neurons in a reversible manner, inducing paralysis, and such STXs are 2000-times more lethal than sodium cyanide by weight [14]. They are classified into several subgroups based on their chemical structure, such as carbamoyl derivatives (STXs, neosaxitoxin (neoSTX), gonyautoxins1–4 (GTX1–4), N-sulphocarbamoyl derivatives (GTX5–6 and C1–4), dicarbamoyl derivatives [dicarbamoyl-STX (dcSTX), dicarbamoyl neosaxitoxin (dcneoSTX), and dicarbamoyl gonyautoxins 1–4 (dcGTX1–4)], and other less-frequent deoxy-decarbonylated, mono-hydroxy-benzoate, di-hydroxy benzoate, and sulphated benzoate analogs [15]. Each compound has a different toxicity and is quantified using the toxicity effect factor (TEF) [16].

In a marine environment, STXs are naturally produced by *Alexandrium* spp., *Gymnodinium catenatum*, and *Pyrodinium bahamense* [17,18,19]. Among them, *Alexandrium* distribute in coastal waters and/or estuary environments, and more than 34 species of the genus have been morphologically identified to date [20,21,22]. Specifically, *Alexandrium catenella*, *Alexandrium pacificum*, and *Alexandrium minutum* are major species causing environmental and industrial damage due to PSTs [23]. Under ideal growth circumstances, some *Alexandrium* grow quickly, generating HABs [13].

HABs of toxic *Alexandrium* can cause paralytic shellfish poisoning (PSP), which can result in illness or mortality in humans via the ingestion of contaminated seafoods [24]. Firstly, shellfish filter-feed toxic dinoflagellates, then the toxins accumulate in the glands of shellfish. In addition, the toxins can be transferred to a variety of marine life, including crabs, starfish, mollusks, turtles, fish, octopus, marine mammals, and seabirds, via bioaccumulation [25]. Therefore, the outbreak of toxic dinoflagellates, especially *Alexandrium*, is a key factor influencing the safety of marine organisms and seafood.

**Figure 2:**
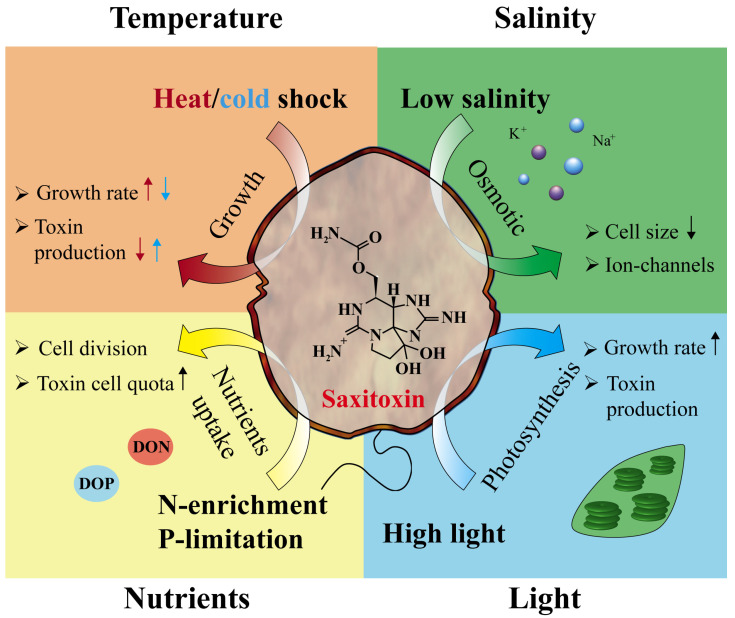
A schematic view of environmental factors that affect cell physiology and saxitoxin: temperature, salinity, nutrients, and light on the physiological metabolism and toxin production of *Alexandrium*. Up and down arrows represent an increase and decrease in the parameters.

Previous studies, including environmental and laboratory, have showed that the proliferation of toxic *Alexandrium* and their STXs production are affected by environmental factors, such as light intensity, temperature, carbon dioxide (CO_2_), salinity, and nutrition [23,26]. Figure 2 represents a schematic view of how major environmental factors affect cell physiology and STXs synthesis in toxic *Alexandrium*. Specifically, water temperature may be more important than nutrients or salinity in determining the abundance of *A. catenella* and the generation of STXs [20]. Environmental investigations and laboratory research imply that temperature may alter the production of STXs in toxic *Alexandrium*, despite the fact that this is contentious [27]. The dynamics of the abiotic variables that drive cell growth and photosynthesis, such as high inorganic nutrient concentrations, optimum temperatures, and light conditions, are not always linked to *Alexandrium* proliferation [28]. Moreover, it is also unclear that STX production is caused by changes in cellular biomass [29].

Toxic *Alexandrium* species are one of the best model organisms for studying STX production, due to their toxicity, wide distribution, and negative impacts on marine habitats. Understanding how environmental conditions influence genetic traits and STXs production in *Alexandrium* is critical [30]. Therefore, this review focused on environmental factors that modulate STXs production by influencing the growth as well as metabolism of STX metabolites and related STX synthesis genes (*sxt*) in toxic *Alexandrium*. In addition, this work will review the insights into STXs production, focusing on the role of several gene complexes in *Alexandrium*.

## 2. Environmental Factors Trigger STXs Production via Altering Growth, Physiology, and Genetic Modulation

Environmental variables, such as nutrients, salinity, temperature, and light intensity, influence the growth of *Alexandrium* cells, of which factors somehow affect STXs production [30,31]. For example, *A. minutum* has a substantially greater cellular toxin capacity when grown in phosphate (PO_4_)-depleted conditions [32]. In addition, temperature and light had effects on the cell growth of *Alexandrium*, and modulated its cellular toxicity [31,33]. The overall production of STXs in relation to environmental factors via altering the growth, physiology, and involvement of genes is detailed below.

### 2.1. Nutrients Modulate the Production of STXs

In terms of nutrition, *Alexandrium* is an opportunistic genus, but one should not expect it to have simple links with conventional nutrients. For instance, *A. catenella* may thrive in both relatively pure and nutrient-rich environments, as well as in waterways that have experienced nutrient abatement [34]. On the contrary, certain concentrations of nutrients, such as carbon (C), nitrogen (N), phosphorus (P), trace metals, and vitamins, also disturb STXs synthesis by *Alexandrium*. Consequently, it is difficult to generalize the nutritional role in *Alexandrium* and its nutrient-dependent mechanisms in relation to HAB formation and PST production. Although the detailed metabolisms are still unknown, nutrient conditions leading to the optimal growth of *Alexandrium* are suspected to influence SXT production. Table 1 summarizes the nutrient conditions for maximum PST production in the genus *Alexandrium* and its toxicity levels.

#### 2.1.1. Carbon (C)

*Alexandrium* species consume dissolved organic carbon (DOC) and dissolved inorganic carbon (DIC) and create oxygen similar to other autotrophs; however, maintenance respiration rates are higher in *Alexandrium* species compared to other phytoplankton genera, such as diatom *Skeletonema costatum* and Ochrophyta *Olisthodiscus luteus* [35]. Interestingly, *Alexandrium* appears to excrete very little DOC, although DIC loss due to respiration is likely to be significant [36]. Depending on the nutritional and physiological state of cells, C fixation seems to be predominantly affected by N availability in *Alexandrium tamarense* [37]. The uncoupling of C and N metabolism is also observed in cultures, with significant increases in C/N ratios following N exhaustion in a time scale of 10 to 17 days [38]. As C is required for *Alexandrium*’s growth and development, it may modulate STXs production. The C-C bond is important for the STXs synthetic process [39]. On the other hand, anthropogenic activities have contributed to the increase in the atmospheric partial pressure of CO_2_ (pCO_2_) altering the dynamics of DIC and changing oceans’ carbonate chemistry [40,41,42]. These factors may affect phytoplankton, which convert inorganic C into organic and secondary metabolites. Previously, Van de Waal et al. (2014) reported that pCO_2_ did not significantly affect the growth and elemental composition of *A. tamarense*; however, increased pCO_2_ reduced PST content [43]. As a result, increased pCO_2_ shifted the PST composition toward more sulfated analogs, thus possibly reducing the cellular toxicity of *A. tamarense* [43]. To date, the toxicity of *Alexandrium* under C-rich or -low environments is still not clear. Hence, further research should be carried out in order to understand STXs production and the genes involved in STXs production in C-rich or -low environments.

**Table 1:** Toxin production in the genus *Alexandrium* under varying nutrient conditions. Saxitoxins equivalent (STXs eq) values were are rounded up to a single digit.

Species	Strain	Nutrient Source	Condition Range (µM/L)	Highest STXs Condition (µM/L)	Toxins	STXs eq (fmol/cell)	Reference
*A. tamarense*	Pr18b	NO_3_	0–880	880	STX, neoSTX, GTX1–4, C1–3	25–49	[44]
	CI01	NO_3_	0–880	880	C2	10–60	[45]
	CI01	PO_4_	5–40	5	C2	20–76	
*A. minutum*	-	PO_4_	13–91	13	STX, GTX1–4	10–27	[32]
	-	NH_4_	40–150	150		29–31	
	-	NO_3_	5–496	496	STX, GTX2, GTX1,4	6–80	[46]
	-	NH_4_	3–230	230		10–19	
*A. pacificum*	HYM9704	NO_3_	50–500	-	neoSTX, GTX1/3, C1–2	15–74	[47]
		PO_4_	1–50	-		68–121	
*A. catenella*	AC11	Fe	0–10	1	GTX1–4	10–89	[48]

#### 2.1.2. Nitrogen (N)

N resources are essential ingredients used to generate nucleic acids, proteins, and other cell components for *Alexandrium* growth and survival [44]. The N/P ratio is also one of the important factors that affects cell size and biovolume. For example, an increase in the N/P ratio (P-limitation) resulted in an increase in the cell sizes of *A. minutum* [44] and *Alexandrium fundyense* [49]. Perhaps, the increase in cell biovolume is induced by arresting cells in the G1 phase without undergoing cell division [50,51], while other non-P compounds are synthesized. Laboratory culture studies have evaluated *Alexandrium*’s growth charges influenced by nitrate (NO_3_), ammonium (NH_4_), and urea availability [52,53,54,55,56]. 

Particularly, NH_4_ contributed a higher N source than NO_3_ for the growth requirements of *A. catenella*, while the differences were not always significant [52]. *Alexandrium* consumes urea and utilizes it to support both cellular physiological and molecular processes in both laboratory and environmental conditions [57]. Growth rate with the supplementation of urea may be lower than that with an NO_3_ or NH_4_ supply. On the other hand, a previous study reported that strains of *A. catenella* and *A. fundyense* did not grow under the condition where urea was supplied as the sole N-source [58]. According to John and Flynn [49], amino-N cannot be used to support the significant growth of *A. fundyense*. Variations in N-dependent growth between strains must be reduced since N availability and their compositions were not always effectively regulated.

##### Total Dissolved Organic Nitrogen (DON)

The effects of dissolved organic nitrogen (DON; >1 kDa) originated from riverine on *A. tamarense* growth in f/2 medium were not significant, despite the fact that chlorophyll contents dropped as the riverine DON level rose [59]. On the other hand, riverine high-molecular-weight (10–100 kDa) DON could benefit *A. minutum* [60]. Similarly, the synergism effects of DON and NO_3_ boosted the growth rate of *A. catenella* by 34% when compared to growth on NO_3_ alone [61]. However, the increased growth rate was not due to NH_4_, suggesting that DON was used directly [61]. Furthermore, in semi-continuous cultures, the effects of marine autotrophic-dissolved organic matter (DOM) on *A. catenella* growth were found to be favorable [61].

##### Nitrate (NO_3_)

Diverse studies have suggested that *Alexandrium* has a linear kinetics of N uptake during NO_3_ assimilation and varies its toxin production [45,62]. The highest toxin content (53.6 fmol/cell) of *A. pacificum* was observed in the NO_3_ replete (1.76 mM) condition [63]. Particularly, an increase in NO_3_ supply continued to increase the toxin yield in *A. tamarense* [45]. The effect of low NO_3_ (88.2 µM NaNO_3_) supplementation on cell biomass and toxin content increased cell density and toxin content by 20–76%, and the toxin value was 43,600 cells/mL [45]. The results show that adding NO_3_ to the cultures at various stages of growth increases the toxin yield by 46% on average [45]. However, continuous low-level NO_3_ supplementation may contribute to an effective increase in *A. tamarense* toxin yield [64]. The NO_3_ absorption systems of *A. catenella* and *A. minutum* have been found to be extremely sensitive to NH_4_ inhibition [65]. 

##### Ammonium (NH_4_)

NH_4_ was discovered to block the urea absorption mechanism in *A. catenella*, but this effect appeared to be strain-dependent [56]. *Alexandrium* species are observed to have unusually high internal levels of arginine and glutamine, which could be precursors to STXs, in different growth periods [66]. In addition, NH_4_ values of 40–150 and 3.0–230 µM/L trigger the production of STXs by 29.2–31.8 and 10–19 fmol/cell in *A. minutum*, respectively [32,46]. At a concentration of 100 µM NH_4_, substrate absorption was inhibited in some *A. tamarense* species [37]. Intra-specific variations in uptake and assimilation kinetics are also significant [62,67]. In addition, NO_3_ triggers the formation of C2 (10–60 fmol/cell) in *A. tamarense* [45]. Despite the extensive research into *Alexandrium* toxicity and toxin-producing activity in N-rich and -deficient settings, the relationship of N and SXTs production cannot be clarified yet, and the results are not consistent and are sometimes controversial. Therefore, further research should be conducted to better understand STXs production and the genes implicated in STXs production in N-rich or -deficient environments.

##### Soil and Bacteria Extracts

The precise mineral or nutrient presence in culture media is required, and soil extracts may help to limit N losses from culture media. In this regard, early research on *Alexandrium* sp. showed that soil extract could boost the growth process compared to completely inorganic media [68]. In addition, soil extracts initiate rapid cell division and promote the rapid growth of the dinoflagellate *P. bahamense* [69]. Humic substances, which result from the biochemical transformation of plant or animal tissues in sediments, have been found to stimulate the growth of *Alexandrium* in a variety of media [68]. Similarly, humic additives considerably increased *A. tamarense* growth rates compared to the controls [70]. Moreover, the addition of humic compounds of riverine origin to an NO_3_-limited medium increased the growth rate of *A. catenella* [71]. However, humic compounds in equimolar concentrations might replace NO_3_ as an N source, while allowing the same species to thrive at equal rates [72].

#### 2.1.3. Phosphorus (P)

Organic P, such as adenosine triphosphate, glycerophosphate, and guanosine diphosphate, can boost the growth rate of some *Alexandrium* species; inorganic P is usually regarded the principal nutrient for *Alexandrium* [58]. Low-molecular-weight organic P appears to be hydrolyzed into inorganic PO_4_ before being used for *A. tamarense* growth [73]. Few studies have looked into inorganic P absorption in *Alexandrium* [74]. In *A. catenella*, the half-saturation of the STXs production of *Alexandrium* constants ranged from 0.01 to 2.6 mM and were shown to be proportional to the growth rate [75]. There have been no reports on multiphasic kinetics, but the concentration range that has been investigated thus far is relatively limited. However, *Alexandrium* appears to be a “storage specialist,” as it can store PO_4_ for future use during periods of P deficiency [76]. A wide range of PO_4_ concentrations resulted in 20.0–76.0 STXs equivalent (STXs eq) fmol/cell of PST in *A. tamarense*, while *A. minutum* produced 10.0–27.5 STXs eq fmol/cell [32]. Furthermore, 1–50 µM/L of PO_4_ increased the STXs production of *A. pacificum* within 24 h and then decreased [47]. N-stress alone resulted in a decrease in toxin per cell, but N-stress followed by P-stress did not, implying that P is involved in the regulation of toxin metabolism [77]. Despite the fact that few investigations on the toxicity and toxin-producing activities of *Alexandrium* in P-rich or -deficient environments have been performed effectively, further research is still necessary to understand STX production and the genes involved in P-rich or -deficient environments.

#### 2.1.4. Miscellaneous

Trace metals, such as selenium, nickel, copper (Cu), cobalt, molybdenum, iron (Fe), manganese, and zinc, are required for algal growth, and they may modulate STXs production in toxic *Alexandrium* [78]. Among those, Fe is the most critical element in dinoflagellate metabolism, including chlorophyll production, electron transport, photosynthesis, and N assimilation [79,80,81]. Specifically, *Alexandrium* has a high Fe requirement for its growth and development. The effect of Fe deficiency on *A. tamarense*, for example, reduced the growth rate and chlorophyll *a* concentration by approximately half [80]. On the other hand, a high concentration of Fe reduced the total toxicity levels to 10.4 fmol/cell (10 µM) compared to 33.6 fmol/cell (1 µM) [80]. This suggests that Fe influences the particular growth rate, cellular biochemical composition, and the synthesis of the toxins in the dinoflagellate *Alexandrium* [48]. In addition, Cu induced toxin production via modulating the growth rate and photosynthetic activity concentration dependently in dinoflagellate *A. minutum* [82]. In addition, metals alter soluble proteomes and toxin profiles in *Alexandrium pacificum* by inhibiting the photosynthetic proteins [78]. Under metal as well as heavy metal stress circumstances, metals modified the STXs profile and soluble proteomes in *A. pacificum*, and such adaptive proteomic responses are related to the development of metal-contaminated ecosystems [78]. However, further studies are required to establish whether trace metals trigger STXs production in toxic *Alexandrium* via modulating the growth.

### 2.2. Temperature: The Most Decisive Factor for STXs Production in Alexandrium

Similar to nutrient conditions, water temperature is a critical environmental factor that affects cell growth and STXs production in both PST-producing cyanobacteria and dinoflagellates [26,29,31,83]. Numerous studies have shown that *Alexandrium* species have different optimal growth temperatures, and even the strains of the same species have varied growth rates and STXs toxicity under identical temperature conditions (Table 2). Overall, a high STXs content was observed during the exponential period under optimal growth conditions [6]. Interestingly, STX synthesis is considerably linked to the growth rate of the species, especially the exponential phase, and therefore, the highest PST levels were mainly analyzed at optimal growth temperatures [26,31,84]. *Alexandrium tamarense*, for example, displayed values in the range of 1.4–2.7 STXs eq fmol/cell and 42.3 STXs eq fmol/cell when cultured at 17 °C and 15 °C, respectively [85,86]. Temperature influences the STXs profile of *A. catenella*, modulating C1 to GTX4 when increasing the temperature from 12 °C to 18–30 °C [87]. Additionally, at 12 °C, C2 dominated at almost all salinities except at 35 and 40 psu, which convert to C3 and C4 [87]. For examples, Ogata et al. (1987) reported that PST production in *A. tamarense* increased with a decrease in growth rates (8 °C), while the increased toxicity at (16 °C) was less noticeable [84]. Similarly, a maximum STXs content was observed at the lowest temperature of 12 °C with moderate growth rates [85]. Also, STXs production in *A. catenella* Alex03 and *A. pacificum* Alex05 significantly increased under cold stress, while it decreased under heat stress [26].

In addition, it was confirmed that not only the total toxicity, but also the composition of the STX analogs, was changed, and the cellular PST levels increased due to an increase in the proportion of toxic compounds with a high TEF [16]. For example, *A. catenella* ACT03 showed a dominance of the C2 toxin when grown at 12–18 °C, while GTX5 became dominant at temperatures in the range of 21–30 °C [87]. Similarly, *A. catenella* Alex03 also mainly produced GTX1 in a range of temperatures (12, 16, and 20 °C), and especially, the absolute concentration of GTX1 (75.4 STXs eq fmol/cell) increased at an optimal growth temperature (16 °C; 86.4 fmol/cell), resulting in about a 4-fold increase in total STXs eq compared to 20 °C [26]. Like *A. pacificum* Alex05, GTX3 and GTX4 were predominantly synthesized at 12, 16, and 20 °C, and the STXs eq considerably increased at 16 °C (64.0 fmol/cell) [31]. Even field surveys showed that total STX levels in scallops contaminated by *A. tamarense* occurred at low temperatures were higher than those at a high temperature [85]. All the results indicate that STXs synthesis is directly affected by temperature and its related processes in *Alexandrium*.

### 2.3. Salinity Modulates STXs Production in Alexandrium 

In the aquatic ecosystem, salinity plays a vital role in controlling organisms’ physiological activities and metabolic processes [95]. Salinity impacts ion concentration or osmotic regulation, which leads to a change in cell size in dinoflagellates [96]. In addition, it also enables the control of intracellular and extracellular enzymes to achieve a stable environment for optimal metabolic activities [97]. Several studies have suggested that *Alexandrium* has species-specific salinity tolerance depending on the geographical origins. For example, Malaysian *A. minutum* displayed high salinity tolerance (5–30 psu) [98], but *A. tamiyavanichii* and *A. tamarense* flourished in a 20–30 psu salinity range [99]. According to Bui et al. (2021), the optimal salinity for *A. pacificum* isolated in Korea was recorded in the range of 30 and 35 psu [30], whereas *A. insuetum* showed maximal growth at 25 psu [100]. These show that different salinity tolerance ranges enable the strain to survive under salinity gradient conditions with different growth patterns [101]. Thus, the effects of salinity on STXs synthesis also varied by research cases, depending on the toxin-producing species and strains [99,102]. Thus, it is still controversial whether low-salinity stress or optimal-salinity conditions have a significant correlation with STXs production in *Alexandrium* [87]. 

The average salinity of marine environments is 33 psu, and diverse laboratory studies have reported that the optimal growth salinity of *Alexandrium* spp. is 25–35 psu. Previous studies also reported that *Alexandrium* tended to produce the highest toxic content under optimal salinity conditions. For example, four different strains of *A. catenella* produced the highest toxin levels ranging from 14.8 to 238.9 STXs eq fmol/cell at 35 psu [102]. Similarly, *A. tamiyavanichii* also peaked in PST contents with optimal growth rates under salinity of 20 and 25 psu [99]. In the case of *A. pacificum*, the highest STXs eq (35.8 fmol/cell) was measured under the optimal condition of 30 psu [98]. Parkhill and Cembella [44] also report that there is a positive correlation between the salinity-dependent growth rate and cellular toxicity of *Alexandrium*, indicating that the PST quota is affected by salinity, like other environmental factors. 

Contrary to the above results, *A. minutum* AmKB06 produced the highest toxin levels (12.0 fmol/cell) at 5 psu, even the optimal growth salinity rates were 15 and 30 psu [99]. In an environmental survey, the PST content of *A. minutum* AM89BM, which occurs in the coast of Brittany (France), was low (10.0 fmol/cell), under 30 to 37 psu, while up to 50.0 fmol/cell was monitored at 15 psu [103]. In contrast, no significant changes in the toxin content were observed when *A. fundyense* experienced short-term exposure to higher and lower salinities [66]. Considering these results, this is still controversial due to the complex relationship between the growth rate, cellular toxin profile, and salinity [84,87].

Apart from total toxicity, salinity also alters the STXs profiles of *Alexandrium*. For example, *A. pacificum* mainly produces GTX4 and C2 at 25–40 psu, while GTX3 is only detected at 20 psu [30]. Hwang and Lu (2000) report that low salinity stimulates *A. minutum* to produce higher amounts of GTX1, while high salinity leads cells to synthesize higher amounts of GTX2–3 [104]. In the case of *A. catenella*, total cellular STXs levels in response to salinity changes were related to changes in C2, GTX4, and GTX5 toxins [87]. In addition, the toxin composition of GTX2+3 was decreased in *A. minutum* with the increase in salinity conditions, and at early and late exponential phases [99]. From these experimental results, we found that the STXs composition of *Alexandrium* changed depending on the salinity conditions and growth stage. Table 3 summarizes the optimal growth conditions and toxin production in the genus *Alexandrium* under different salinity conditions from the published literature.

Variations in membrane transport routes are one of the initial cell reactions to salinity changes, causing metabolic functions to be modified [95]. Extreme salinity conditions may limit the production of STXs by altering arginine synthesis, the primary precursor of STX biosynthesis [98]. It consequently decreases the activity of ornithine, glutamine, arginine, and carbamoyl phosphate synthesis metabolism [105]. To date, the toxin-producing activity of *Alexandrium* in various salt environments is still unclear; hence, more research should be carried out to understand STXs production in toxic dinoflagellates.

**Table 3:** Optimal growth conditions and saxitoxins (STXs) production in the genus *Alexandrium* under different salinity conditions from the published literature. STXs equivalent (STXs eq) values are rounded up to a single digit.

Species	Strain	Origin	Salinity Range	Optimal Growth Salinity	Highest STXs Condition	Toxins	STXs eq (fmol/cell)	Reference
*A. catenella*	PFB38	Chile	15–35	35	35	neoSTX, GTX1–5	95	[102]
	ACT03	Mediterranean Sea	10–40	30	35	C1–4, GTX3–5	50	[87]
*A. fundyense*	MI	USA	15–35	25	30	STX, neoSTX, GTX1–4	62	[91]
	BoF	USA	15–35	25	30–35	STX, neoSTX, GTX1–4	73–75	
*A. minutum*	AM89BM	France	12–37	20–37	15	-	50	[103]
	AmKB06	Malaysia	2–30	15	5	GTX1–6, C2, neoSTX, dcSTX	12	[99]
	Alexsp17	Vietnam	5–35	10–15	30–35	STX, neoSTX, dcSTX, C2, GTX2–4	30	[98]
*A. ostenfeldii*	AOSH1	Canada	15–33	33	15	C3	−	[33]
	OKNL21	Netherlands	3–34	22	5	STX, GTX2/3/5, C1–2	52	[106]
*A. peruvianum*	ApKS01	Malaysia	2–30	25	25	GTX1–6, C2, neoSTX, dcSTX	0.8	[99]
*A. pacificum*	Alex05	Republic of Korea	20–40	30	30	neoSTX, dcSTX, dcGTX2, STX, GTX1–6, C1–2	36	[30]
*A. tamarense*	Pr18b	Canada	10–30	25	25	STX, neoSTX, GTX1–4, C1–3	179	[44]
	AtPA01	Malaysia	2–30	20–30	15	GTX1–6, C2, neoSTX, dcSTX	0.8	[99]
*A. tamiyavanichii*	AcMS01	Malaysia	2–30	25	20	GTX1–6, C2, neoSTX, dcSTX	80	[99]

### 2.4. Light Intensity: The Crucial Factor for the Growth of Alexandrium and STXs Production

Until now, STXs have been identified as being produced by photosynthetic cyanobacterium, such as *Dolichospermum* spp., *Raphidiopsis raciborskii*, and dinoflagellates, like *Alexandrium* spp. and *G. catenatum* [107]. Thus, the direct and indirect effects of light on STXs production are considerable and complex. Previous studies have already shown that photosynthetic activity affects STXs synthesis [44,84]. In detail, STXs biosynthesis requires the use of additional C skeletons produced during photosynthesis, such as amino acids and acetate, as well as high-energy light intermediates, such as ATP and NADH/NADPH [108]. Such amino acids are produced during short periods of photo-assimilation [109], and this also requires incorporated NO_3_ [110,111]. Therefore, the decrease in light intensity can suppress the fresh synthesis of amino acids and N assimilation [84], affecting growth and varied metabolisms, including the production of secondary metabolites. 

Photo-assimilation plays an important role in the production of toxins by the dinoflagellate *A. tamarense* [84]. The photo-assimilation of NO_3_ or ammonia (NH_3_) into amino acid precursors may be related to the production of STXs requiring abundant N sources [63,84]. In this regard, the intensity of light controls *Alexandrium* growth rate and STXs synthesis, where lowering the intensity of light increases the toxicity, while decreasing growth increases and vice versa [84]. Contrary to this, Parkhill and Cembella [44] reported that changes in PST levels were largely independent of light, but depended on the growth stages, suggesting that light did not provide a direct response to STXs synthesis. Various other studies have found that *Alexandrium* species respond differently to the increase or decrease in PST levels depending on light strength [85,91,92,104]. Table 4 summarizes the optimal growth conditions and toxin production in the genus *Alexandrium* under different light intensities from the published literature. 

The amount of light passing through water and its spectral quality are both important factors for determining the photosynthetic rate of dinoflagellates [112]. Since water selectively absorbs and scatters white light, the intensity and spectrum quality of light vary significantly depending on its turbidity and depth. Several dinoflagellates have a meroplanktonic life cycle and migrate vertically from nutrient-depleted and light-rich surface water to dark but nutrient-rich water, facing varied light intensities and spectral-light characteristics [113]. Their plastids are unique among photosynthetic alveolates, since they contain the light-harvesting system based on peridinin–chlorophyll–protein (PCP) [114]. When irradiance is inhibited, dinoflagellate *Glenodinium* sp. increases photon capture and maintains photosynthetic efficiency by increasing the pigment molecules linked to the reaction centers in chloroplasts [115]. As such, the quality of light affects the algal photosynthesis rate, which is very important since it affects cellular metabolism, such as pigment composition, nutrient and C uptake, and even toxin synthesis [116].

Many studies have reported that light intensity and spectrum quality have a significant effect on the synthesis of various toxins and secondary metabolites of microalgae [117,118]. In the case of toxic dinoflagellates, especially *Alexandrium* spp., STXs production is also influenced by light quality. For example, *A. tamarense* displayed the highest toxin production at 150 μmol photons/m^2^/s and the amount was 0.93–5.9 fmol/cell [44]. The toxins (neoSTX, GTX1–6, and C1–2) produced in *A. catenella* at 100 μmol photons/m^2^/s are recorded at 150–350 STXs eq fmol/cell [119]. Additionally, *A. catenella* ACT03 produced toxins like GTX3–5, C2/4 in a range of 13.7–24.7 STXs eq fmol/cell at 260 μmol photons/m^2^/s [87]. Moreover, in *A. fundyense*, the highest toxin production rates were 20–100 and 60–150 STXs eq fmol/cell at 175 and 425 μmol photons/m^2^/s, respectively [91]. In addition, *A. tamiyavanichii* produced 60.0–180.0 fmol STXs eq/cell (STX, GTX1–5, C2, dcSTX) at light range of 10–100 μmol photons/m^2^/s [92]. In addition, *A. minutum* displayed GTX1, GTX4 production values of 10–42 fmol/cell at a light range of 10–100 μmol photons/m^2^/s [92]. At 100 μmol photons/m^2^/s, *A. pacificum* produced 800–1400 STXs eq fmol/cell, and this is the maximum production of STX [119]. Overall, these findings show that light plays a crucial role in algal growth and cellular metabolism, thereby affecting PST production in *Alexandrium*.

**Table 4:** Optimal growth conditions and saxitoxins (STXs) production in the genus *Alexandrium* under different irradiance conditions from the published literature. STXs equivalent (STXs eq) values are rounded up to a single digit.

Species	Strain	Light Range (μmol Photons/m^2^/s)	Optimal Growth Condition	Highest STXs Condition	Toxins	STXs eq (fmol/cell)	Reference
*A. tamarense*	Pr18b	40–470	230	150	STX, neoSTX, GTX1–4, C1–3	0.9–6	[44]
*A. catenella*	ACT03	10–260	−	260	GTX3–5, C2/4	14–25	[87]
	KNU-YS-01	10–300	300	100	neoSTX, GTX1–6, C1–2	150–350	[119]
*A. fundyense*	MI	6–425	425	175	STX, neoSTX, GTX1–4	20–100	[91]
	BoF	6–425	425	425	STX, neoSTX, GTX1–4	60–150	
*A. tamiyavanichii*	AcMS01	10–100	100	>50	STX, GTX1–5, C2, dcSTX	60–180	[92]
*A. minutum*	AmKB02	10–100	100	>24	GTX1/4	10–42	[92]
*A. pacificum*	LIMS-PS-2792	10–300	200	100	GTX1–6, C1–2, dcGTX2–3	800–1400	[119]

## 3. Adaptation Mechanism of *Alexandrium* in Response to Environmental Conditions

Organisms respond differently to different environmental drivers, mostly as a result of metabolic disruptions [120]. Moreover, it can be expected that different stressors affect the metabolic pathways responsible for the production of various toxin groups [120]. Despite intensive efforts to understand the effects of environmental conditions on STX synthesis in diverse *Alexandrium* species, it still remains difficult to predict how these harmful dinoflagellates will respond to abiotic factors and result in toxicity changes. Moreover, reports on the factor interactions of multiple environmental conditions often contradict each other, and thus form a complex picture that is difficult to explain [13]. Other reports on the STXs production of *Alexandrium* in relation to such abiotic factors also highlight this problem [23,94]. As described previously, several abiotic factors obviously affect *Alexandrium* growth and its toxicity. As well as abiotic factors, genetic factors, like phenotypic variability, have long been recognized in phytoplankton, and have recently become the focus of significant research in the genus *Alexandrium* [23]. Molecular research has revealed that *Alexandrium* populations have a lot of genetic variations [23,121]. Such variations are expected to represent the adaptation of the population to environmental and climate changes [122]. Climate factors control the diversity within a particular species, and each population has different preferred temperature, salinity, pCO_2_, and nutrient conditions [123]. For all parameters, significant effects of genotype on the response to temperature and salinity changes were identified [124]. For example, experiments testing the responses of varied strains under predicted changes in environmental conditions by the end of the century have been performed to better characterize the consequences of potential genotype shifts in relation to climate change in the future [123]. These findings suggest that the adaptation to abiotic factors is aided by altering the genetic variation.

## 4. Genetic Understanding of Environmental Factors and STXs Biosynthesis *sxt* Genes

**Figure 3:**
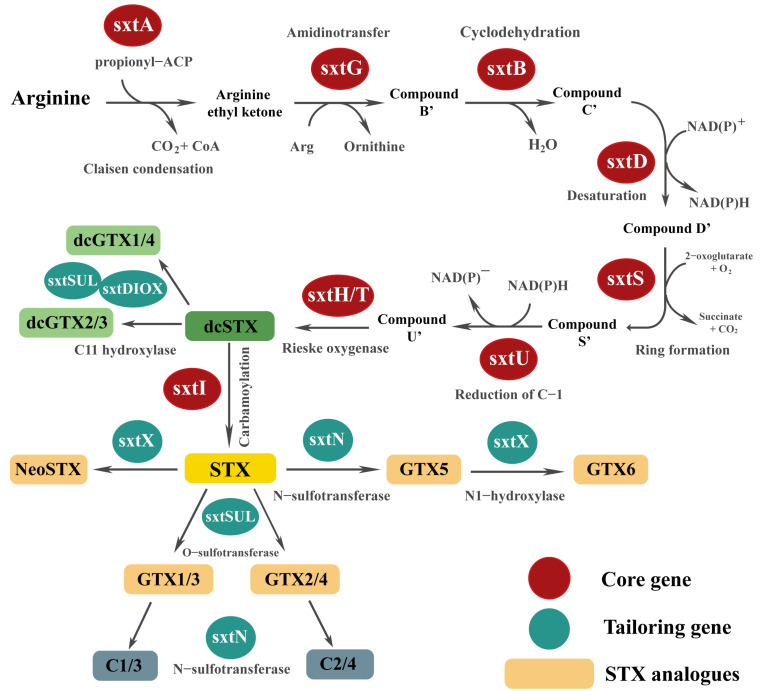
PST biosynthetic pathway in dinoflagellates. The *sxt* core and tailoring genes are in red and blue circles. The STX analogs are in colored rectangles. Gray indicates added groups catalyzed by each enzyme. ACP, acyl carrier protein; CoA, malonyl-CoA; Arg, arginine; NAD(P)^+^/NAD(P)H, oxidized/reduced forms of nicotinamide adenine dinucleotide; STX, saxitoxin; dcSTX, decarbamoyl-saxitoxin; GTXs, gonyautoxins; neoSTX, neosaxitoxin; C, N-sulfocarbamoyl-saxitoxin. Modified from Kellmann et al. [125], Mihali et al. [137,138], and Cullen et al. [139].

STX is biochemically synthesized by the stepwise involvement of eight core enzymes following catalytic reactions, as seen in Figure 3 [125,126]. The biochemical studies of PSTs started with STX, which was first isolated from the Alaskan butter clam *Saxidomus giganteus* in 1957 [127]. Additionally, the structure of STX was also discovered in the 1980s in a PST-producing cyanobacterium, *R. raciborskii* T3, and the candidate gene (*sxt*) cluster for the PST biosynthesis pathway was identified. Twenty-six proteins grouped in a single 35 kb cluster have 30 catalytic functions [125,126]. Among these, eight enzymes (sxtA, sxtB, sxtD, sxtG, sxtS, sxtH/T, sxtU, and sxtI) are directly involved in STX synthesis in cyanobacteria [125,126,128,129]. Moreover, genes participating in the tailoring, regulation, and transportation of STXs are involved in synthesizing various PST analogs [130,131,132,133]. Similarly, toxic dinoflagellates have *sxt* gene homologs to cyanobacteria [12,134,135,136].

Among the genes, *sxtA* is known to be involved in the initial stage of STX synthesis and is the most widely studied [140]. Cyanobacterial sxtA includes the N-terminal and C-terminal, while dinoflagellate sxtA contains four catalytic domains (sxtA1, sxtA2, sxtA3, and sxtA4) [128]. The single mRNA candidate of *sxtA* was found in cyanobacteria, while more than two *sxtA* isoforms were found in toxic dinoflagellates *A. fundyense*, *A. minutum*, *A. catenella*, and *A. pacificum* through RNA-sequencing analysis [141,142]. These consist of “sxtA short form” that encodes three catalytic domains (sxtA1–3) and a “sxtA long form” that encodes four catalytic domains (sxtA1–4) [141]. A recent study revealed that sxtA1–3 domains were stably present in bacteria, cyanobacteria, and dinoflagellates, but the sxtA long form was only found in STX-producing cyanobacteria and dinoflagellates [107,135]. Through various molecular studies, *sxtA4* was identified in various toxic dinoflagellates, including *Alexandrium* spp., *G. catenatum*, and *P. bahamense*. Furthermore, it was suggested that the presence of *sxtA4* and its copy number have a significant relationship with STX synthesis [9,134].

Further genetic studies were also conducted focusing on the secondary core *sxtG* gene, which catalyzes the incorporation of the amidino group from the product of sxtA in the STX synthesis process [141,143]. Similar to *sxtA*, the loss and/or modification of the gene results in the breakdown of the initial reaction in STX production, and it may lead to a loss of the ability of toxin production in both cyanobacteria and dinoflagellates [144]. In addition to this, the existence and mutation of other *sxt* genes were also linked to PST production [31,144]. As a result, *sxt* gene structure and expressional regulations are two critical aspects to investigate in order to properly comprehend PST synthesis processes in dinoflagellates, especially *Alexandrium*. 

**Figure 4:**
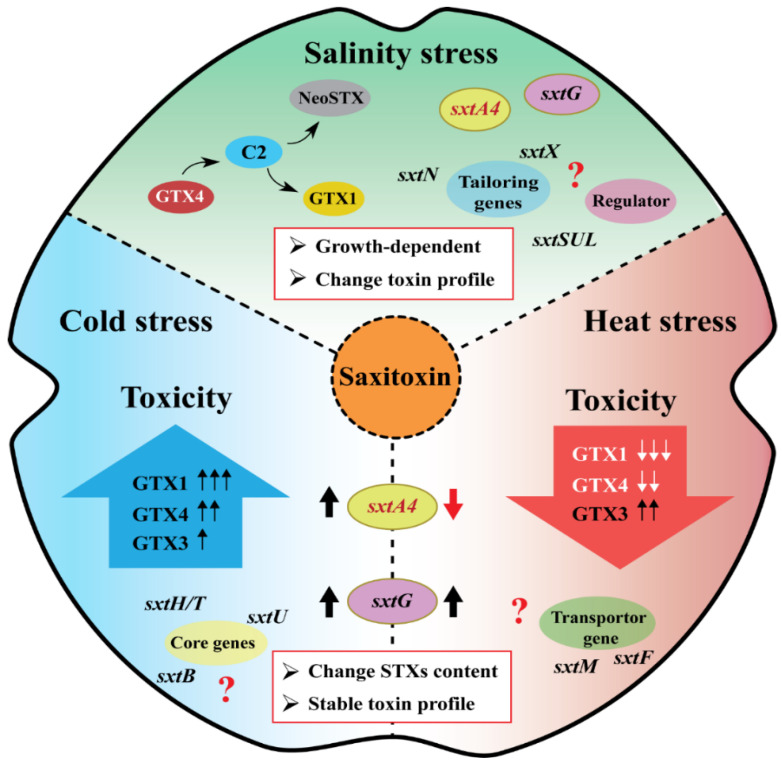
A schematic representation of the STXs production pathway and gene regulations in toxic *Alexandrium* under temperature and salinity stresses. Thick up and down arrows represent an increase and decrease in parameters.

In general, the cell size changes according to the cell division rate, which may alter cytotoxicity in toxic dinoflagellates [145]. Therefore, it was necessary to determine whether environmental factors induce STXs production or simply change cellular toxicity depending on the cell size. To understand the alternation in STXs synthesis from a molecular perspective, core *sxt* genes, especially *sxtA* and *sxtG*, were identified in toxic *Alexandrium*, and their transcription patterns were evaluated under different environmental conditions (Figure 4). In summary, the expression patterns of *sxtA* and *sxtG* and their correlation with PST production were different under salinity and water temperature conditions.

Temperature, especially cold stress and optimal conditions, induced both cellular STXs levels and the two core gene expression levels in the toxic dinoflagellates *A. catenella* Alex03 and *A. pacificum* Alex05 [26,31]. On the other hand, the gene expression levels decreased under high-salinity conditions (40 psu), and there was no significant relationship between cellular STXs toxicity in *A. pacificum* [30]. Instead, the author suggested that STX tailoring genes (*sxtX* and *sxtN*) may be involved in the structural conversion from STX to other derivatives, including GTXs and dcGTXs. Besides *sxtA* and *sxtG*, recent studies reported the full-length sequences of *sxtB* [136], *sxtU* [146], and *sxtI* [63] from *Alexandrium* spp., and showed the correlation between their expression, toxin production, and environmental changes. Since each STXs metabolite has a different TEF, affecting the total STXs eq [16], it is contradictory to interpret the reduction in STXs eq as a decrease that is an absolute reduction. Therefore, further research is needed to understand the entire transcriptional response of *sxt* that relates to STXs production (*sxtB*, *sxtD*, *sxtI*, *sxtS*, and *sxtU*), modification (*sxtL*, *sxtN*, and *sxtX*), and transport (*sxtF* and *sxtM*), which alter the toxins’ composition [139]. Additionally, other environmental factors, such as nutrients and light, may influence STXs biosynthesis genes and PST production in dinoflagellates. Hence, more research should focus on the genes responsible for STXs synthesis in *Alexandrium* under different abiotic stress conditions.

## 5. Conclusions and Future Prospects

The cell growth and STXs production of toxic *Alexandrium* are obviously affected by environmental conditions, including nutrients, salinity, temperature, and light intensity. Recent studies on the expression and regulation of *sxt* shed light on the influence of abiotic factors on toxin production in diverse strains of *Alexandrium*. However, molecular research on *sxt* and PST synthesis in *Alexandrium* is rare, and no definite conclusion has yet been reached. Therefore, further research should be carried out to understand the molecular mechanism of STXs production based on entire transcriptome analysis under diverse environmental conditions. Multi-omcis-based studies, especially transcriptomic and metabolomic approaches, are considered as an alternative to assess the large-scale molecular mechanisms of dinoflagellates due to their extraordinary genomic features. Analyses of intracellular metabolic changes under varied environmental conditions, such as eutrophication, acidification, and increase in water temperature, allowed us to understand the mechanisms of HABs and PST outbreaks. In this regard, finding molecular detection research on the selected biomarkers highly relevant to STXs synthesis will provide us with information to predict and manage PSP occurrences. Finally, computational models that compute the interaction between abiotic factors and physiological and molecular influences can help us better forecast the global changes in *Alexandrium* proliferation patterns and PST production conditions in marine ecosystems.

## Figures and Tables

**Table 2:** Saxitoxins (STXs) production in the genus *Alexandrium* under different temperature conditions from the published literature. STXs equivalent (STXs eq) values are rounded up to a single digit.

Species	Strain	Origin	Temperature	Toxins	STXs eq (fmol/cell)	Reference
*A. catenella*	ACC02	Chile	10–16 °C	−	3–75	[88]
	ATTL01	France	15 °C	−	0.2	[89]
	Alex03	Republic of Korea	12–20 °C	neoSTX, dcSTX, dcGTX2, STX, GTX1–6, C1–2	15–97	[26]
	CCAP1119/27	Scotland	15 °C	−	9	[90]
*A. fundyense*	BOF	USA	5–20 °C	STX, neoSTX, GTX1–4	211–544	[91]
	MI	USA	5–20 °C	STX, neoSTX, GTX1–4	100–532	
*A. lusitanicum*	BAH91	Portugal	15 °C	GTX1–4	16	[86]
	AL3T	Gulf of Trieste	15 °C	GTX1–4	3	
*A. minutum*	AmSp01	Vietnam	25 ℃	neoSTX, GTX1/3/4	11–13	[92]
	AmSp03	Vietnam	25 ℃	neoSTX, GTX1/4	9–12	
	AmSp04	Vietnam	25 ℃	neoSTX, GTX1/3/4	5–11	
	AmSp05	Vietnam	25 ℃	dcSTX, neoSTX, GTX1–4	3–10	
	AmSp17	Vietnam	25 ℃	dcSTX, neoSTX, GTX1/3/4	5	
*A. tamarense*	ATHS–95	Japan	17 °C	C1–2, GTX1–4	1–3	[85]
	BAH181	Orkney Island	15 °C	neoSTX, STX, B1–2, C1–2, GTX1–4	42	[86]
	GTPP01	USA	15 °C	neoSTX, STX, B1–2, C1–2, GTX1–4	33	
*A. pacificum*	ANG4–4	Algerian	20 °C	neoSTX, STX, C2, GTX4/6	4	[93]
	Alex05	Republic of Korea	12–20 °C	neoSTX, dcSTX, dcGTX2, STX, GTX1–6, C1–2	0.3–132	[31]
	IFR-ACA-15	Mediterranean Sea	17 °C	C1–2, GTX4–5, dcGTX2	10–22	[94]

## Data Availability

Not applicable.

## References

[1] Pradhan B., Maharana S., Bhakta S., Jena M. (2021). Marine phytoplankton diversity of Odisha coast, India with special reference to new record of diatoms and dinoflagellates. Vegetos.

[2] Behera C., Pradhan B., Panda R., Nayak R., Nayak S., Jena M. (2021). Algal Diversity of Saltpans, Huma (Ganjam), India. J. Indian Bot. Soc..

[3] Dash S., Pradhan B., Behera C., Nayak R., Jena M. (2021). Algal flora of Tampara lake, Chhatrapur, Odisha, India. J. Indian Bot. Soc..

[4] Dash S., Pradhan B., Behera C., Jena M. (2020). Algal diversity of Kanjiahata lake, Nandankanan, Odisha, India. J. Indian Bot. Soc..

[5] Behera C., Dash S.R., Pradhan B., Jena M., Adhikary S.P. (2020). Algal diversity of Ansupa lake, Odisha, India. Nelumbo.

[6] Reich A., Lazensky R., Faris J., Fleming L.E., Kirkpatrick B., Watkins S., Ullmann S., Kohler K., Hoagland P. (2015). Assessing the impact of shellfish harvesting area closures on neurotoxic shellfish poisoning (NSP) incidence during red tide (*Karenia brevis*) blooms. Harmful Algae.

[7] Pierella Karlusich J.J., Ibarbalz F.M., Bowler C. (2020). Phytoplankton in the Tara ocean. Ann. Rev. Mar. Sci..

[8] Taylor F., Hoppenrath M., Saldarriaga J.F. (2008). Dinoflagellate diversity and distribution. Biodivers. Conserv..

[9] Wang H., Guo R., Lim W.-A., Allen A.E., Ki J.-S. (2020). Comparative transcriptomics of toxin synthesis genes between the non-toxin producing dinoflagellate *Cochlodinium polykrikoides* and toxigenic *Alexandrium pacificum*. Harmful Algae.

[10] Pradhan B., Kim H., Abassi S., Ki J.-S. (2022). Toxic effects and tumor promotion activity of marine phytoplankton toxins: A Review. Toxins.

[11] Pradhan B., Ki J.-S. (2022). Phytoplankton toxins and their potential therapeutic applications: A journey toward the quest for potent pharmaceuticals. Mar. Drugs.

[12] Stüken A., Orr R.J., Kellmann R., Murray S.A., Neilan B.A., Jakobsen K.S. (2011). Discovery of nuclear-encoded genes for the neurotoxin saxitoxin in dinoflagellates. PLoS ONE.

[13] Anderson D.M., Alpermann T.J., Cembella A.D., Collos Y., Masseret E., Montresor M. (2012). The globally distributed genus *Alexandrium*: Multifaceted roles in marine ecosystems and impacts on human health. Harmful Algae.

[14] Cestèle S., Catterall W.A. (2000). Molecular mechanisms of neurotoxin action on voltage-gated sodium channels. Biochimie.

[15] Wiese M., D’agostino P.M., Mihali T.K., Moffitt M.C., Neilan B.A. (2010). Neurotoxic alkaloids: Saxitoxin and its analogs. Mar. Drugs.

[16] EFSA (2009). Marine biotoxins in shellfish–Saxitoxin group, scientific opinion of the panel on contaminants in the food chain. EFSA J..

[17] Deeds J.R., Landsberg J.H., Etheridge S.M., Pitcher G.C., Longan S.W. (2008). Non-traditional vectors for paralytic shellfish poisoning. Mar. Drugs.

[18] Oshima Y., Sugino K., Itakura H., Hirota M., Yasumoto Y., Granelli E., Sundstrom B., Edler L., Anderson D.M. (1990). Comparative studies on paralytic shellfish toxin profile of dinoflagellates and bivalves. Toxic Marine Phytoplankton.

[19] Anderson D.M., Sullivan J.J., Reguera B. (1989). Paralytic shellfish poisoning in northwest Spain: The toxicity of the dinoflagellate *Gymnodinium catenatum*. Toxicon.

[20] Vandersea M.W., Kibler S.R., Tester P.A., Holderied K., Hondolero D.E., Powell K., Baird S., Doroff A., Dugan D., Litaker R.W. (2018). Environmental factors influencing the distribution and abundance of *Alexandrium catenella* in Kachemak bay and lower cook inlet, Alaska. Harmful Algae.

[21] Condie S.A., Oliver E.C., Hallegraeff G.M. (2019). Environmental drivers of unprecedented *Alexandrium catenella* dinoflagellate blooms off eastern Tasmania, 2012–2018. Harmful Algae.

[22] Guiry M., Guiry G. AlgaeBase. World-Wide Electronic Publication, National University of Ireland, Galway. http://www.algaebase.org.

[23] Murray S., John U., Savela H., Kremp A., Botana L.M., Louzao M.C. (2015). *Alexandrium* spp.: Genetic and ecological factors influencing saxitoxin production and proliferation. Climate Change and Marine and Freshwater Toxins.

[24] Etheridge S.M. (2010). Paralytic shellfish poisoning: Seafood safety and human health perspectives. Toxicon.

[25] Llewellyn L., Negri A., Robertson A. (2006). Paralytic shellfish toxins in tropical oceans. Toxin Rev..

[26] Kim H., Park H., Wang H., Yoo H.Y., Park J., Ki J.-S. (2021). Low temperature and cold stress significantly increase saxitoxins (STXs) and expression of STX biosynthesis genes *sxtA4* and *sxtG* in the dinoflagellate *Alexandrium catenella*. Mar. Drugs.

[27] Eckford-Soper L.K., Bresnan E., Lacaze J.-P., Green D.H., Davidson K. (2016). The competitive dynamics of toxic *Alexandrium fundyense* and non-toxic *Alexandrium tamarense*: The role of temperature. Harmful Algae.

[28] Gettings R.M., Townsend D.W., Thomas M.A., Karp-Boss L. (2014). Dynamics of late spring and summer phytoplankton communities on Georges Bank, with emphasis on diatoms, *Alexandrium* spp., and other dinoflagellates. Deep Sea Res. Part II Top. Stud. Oceanogr..

[29] Cirés S., Delgado A., González-Pleiter M., Quesada A. (2017). Temperature influences the production and transport of saxitoxin and the expression of *sxt* genes in the cyanobacterium *Aphanizomenon gracile*. Toxins.

[30] Bui Q.T.N., Kim H., Park H., Ki J.-S. (2021). Salinity affects saxitoxins (STXs) toxicity in the dinoflagellate *Alexandrium pacificum*, with low transcription of SXT-biosynthesis genes *sxtA4* and *sxtG*. Toxins.

[31] Wang H., Kim H., Park H., Ki J.-S. (2022). Temperature influences the content and biosynthesis gene expression of saxitoxins (STXs) in the toxigenic dinoflagellate *Alexandrium pacificum*. Sci. Total Environ..

[32] Hii K.S., Lim P.T., Kon N.F., Takata Y., Usup G., Leaw C.P. (2016). Physiological and transcriptional responses to inorganic nutrition in a tropical Pacific strain of *Alexandrium minutum*: Implications for the saxitoxin genes and toxin production. Harmful Algae.

[33] Maclean C., Cembella A.D., Quilliam M.A. (2003). Effects of light, salinity and inorganic nitrogen on cell growth and spirolide production in the marine dinoflagellate *Alexandrium ostenfeldii* (Paulsen) Balech et Tangen. Bot. Mar..

[34] Collos Y., Bec B., Jauzein C., Abadie E., Laugier T., Lautier J., Pastoureaud A., Souchu P., Vaquer A. (2009). Oligotrophication and emergence of picocyanobacteria and a toxic dinoflagellate in Thau lagoon, southern France. J. Sea Res..

[35] Langdon C. (1987). On the causes of interspecific differences in the growth-irradiance relationship for phytoplankton. Part I. A comparative study of the growth-irradiance relationship of three marine phytoplankton species: *Skeletonema costatum*, *Olisthodiscus luteus* and *Gonyaulax tamarensis*. J. Plankton Res..

[36] Flynn K.J., Clark D.R., Xue Y. (2008). Modeling the release of dissolved organic matter by phytoplankton. J. Phycol..

[37] Leong S.C.Y., Maekawa M., Taguchi S. (2010). Carbon and nitrogen acquisition by the toxic dinoflagellate *Alexandrium tamarense* in response to different nitrogen sources and supply modes. Harmful Algae.

[38] Flynn K., Jones K., Flynn K. (1996). Comparisons among species of *Alexandrium* (*Dinophyceae*) grown in nitrogen-or phosphorus-limiting batch culture. Mar. Biol..

[39] Paladugu S.R., James C.K., Looper R.E. (2019). A direct C11 alkylation strategy on the saxitoxin core: A synthesis of (+)-11-saxitoxinethanoic acid. Org. Lett..

[40] Tortell P.D., Payne C.D., Li Y., Trimborn S., Rost B., Smith W.O., Riesselman C., Dunbar R.B., Sedwick P., DiTullio G.R. (2008). CO_2_ sensitivity of Southern Ocean phytoplankton. Geophys. Res. Lett..

[41] Kaushal S.S., Duan S., Doody T.R., Haq S., Smith R.M., Johnson T.A.N., Newcomb K.D., Gorman J., Bowman N., Mayer P.M. (2017). Human-accelerated weathering increases salinization, major ions, and alkalinization in fresh water across land use. Appl. Geochem..

[42] Raven J.A., Gobler C.J., Hansen P.J. (2020). Dynamic CO_2_ and pH levels in coastal, estuarine, and inland waters: Theoretical and observed effects on harmful algal blooms. Harmful Algae.

[43] Van de Waal D.B., Eberlein T., John U., Wohlrab S., Rost B. (2014). Impact of elevated pCO_2_ on paralytic shellfish poisoning toxin content and composition in *Alexandrium tamarense*. Toxicon.

[44] Parkhill J.-P., Cembella A.D. (1999). Effects of salinity, light and inorganic nitrogen on growth and toxigenicity of the marine dinoflagellate *Alexandrium tamarense* from northeastern Canada. J. Plankton Res..

[45] Wang D.-Z., Hsieh D.P. (2002). Effects of nitrate and phosphate on growth and C2 toxin productivity of *Alexandrium tamarense* CI01 in culture. Mar. Pollut. Bull..

[46] Lim P.-T., Leaw C.-P., Kobiyama A., Ogata T. (2010). Growth and toxin production of tropical *Alexandrium minutum* Halim (*Dinophyceae*) under various nitrogen to phosphorus ratios. J. Appl. Phycol..

[47] Han M., Lee H., Anderson D.M., Kim B. (2016). Paralytic shellfish toxin production by the dinoflagellate *Alexandrium pacificum* (Chinhae Bay, Korea) in axenic, nutrient-limited chemostat cultures and nutrient-enriched batch cultures. Mar. Pollut. Bull..

[48] Yarimizu K., Mardones J.I., Paredes-Mella J., Norambuena-Subiabre L., Carrano C.J., Maruyama F. (2022). The effect of iron on Chilean *Alexandrium catenella* growth and paralytic shellfish toxin production as related to algal blooms. BioMetals.

[49] John E., Flynn K. (2000). Growth dynamics and toxicity of *Alexandrium fundyense* (*Dinophyceae*): The effect of changing N/P supply ratios on internal toxin and nutrient levels. Eur. J. Phycol..

[50] Juhl A.R., Latz M.I. (2002). Mechanisms of fluid shear—Induced inhibition of population growth in a red-tide dinoflagellate. J. Phycol..

[51] Yeung P.K.K., Wong J.T.Y. (2003). Inhibition of cell proliferation by mechanical agitation involves transient cell cycle arrest at G1 phase in dinoflagellates. Protoplasma.

[52] Dyhrman S.T., Anderson D.M. (2003). Urease activity in cultures and field populations of the toxic dinoflagellate *Alexandrium*. Limnol. Oceanogr..

[53] Huang K., Feng Q., Zhang Y., Ou L., Cen J., Lu S., Qi Y. (2020). Comparative uptake and assimilation of nitrate, ammonium, and urea by dinoflagellate *Karenia mikimotoi* and diatom *Skeletonema costatum* s.l. in the coastal waters of the East China Sea. Mar. Pollut. Bull..

[54] Chang F.H., McClean M. (1997). Growth responses of *Alexandrium minutum* (*Dinophyceae*) as a function of three different nitrogen sources and irradiance. N. Z. J. Mar. Freshw. Res..

[55] Shankar S., Townsend D.W., Thomas M.A. (2014). Ammonium and maintenance of bloom populations of *Alexandrium fundyense* in the Gulf of Maine and on Georges Bank: Results of laboratory culture experiments. Mar. Ecol. Prog. Ser..

[56] Jauzein C., Loureiro S., Garcés E., Collos Y. (2008). Interactions between ammonium and urea uptake by five strains of *Alexandrium catenella* (*Dinophyceae*) in culture. Aquat. Microb. Ecol..

[57] Collos Y., Vaquer A., Laabir M., Abadie E., Laugier T., Pastoureaud A., Souchu P. (2007). Contribution of several nitrogen sources to growth of *Alexandrium catenella* during blooms in Thau lagoon, southern France. Harmful Algae.

[58] Matsuda A., Nishijima T., Fukami K. (1999). Effects of nitrogenous and phosphorus nutrients on the growth of toxic dinoflagellate *Alexandrium catenella*. Bull. Jpn. Soc. Sci. Fish..

[59] Stolte W., Panosso R., Gisselson L.-Å., Granéli E. (2002). Utilization efficiency of nitrogen associated with riverine dissolved organic carbon (> 1 kDa) by two toxin-producing phytoplankton species. Aquat. Microb. Ecol..

[60] Fagerberg T., Carlsson P., Lundgren M. (2009). A large molecular size fraction of riverine high molecular weight dissolved organic matter (HMW DOM) stimulates growth of the harmful dinoflagellate *Alexandrium minutum*. Harmful Algae.

[61] Loureiro S., Garcés E., Collos Y., Vaqué D., Camp J. (2009). Effect of marine autotrophic dissolved organic matter (DOM) on *Alexandrium catenella* in semi-continuous cultures. J. Plankton Res..

[62] Jauzein C., Collos Y., Garcés E., Vila M., Maso M. (2008). Short-term temporal variability of ammonium and urea uptake by *Alexandrium catenella* (Dinophyta) in cultures. J. Phycol..

[63] Abassi S., Kim H.S., Bui Q.T.N., Ki J.S. (2023). Effects of nitrate on the saxitoxins biosynthesis revealed by *sxt* genes in the toxic dinoflagellate *Alexandrium pacificum* (group IV). Harmful Algae.

[64] Hu H., Chen W., Shi Y., Cong W. (2006). Nitrate and phosphate supplementation to increase toxin production by the marine dinoflagellate *Alexandrium tamarense*. Mar. Pollut. Bull..

[65] Maguer J.F., l’Helguen S., Madec C., Labry C., Le Corre P. (2007). Nitrogen uptake and assimilation kinetics in *Alexandrium minutum* (*Dinophyceae*): Effecr of N–limited growth rate on nitrate and ammonium interaction. J. Phycol..

[66] Anderson D., Kulis D., Sullivan J., Hall S., Lee C. (1990). Dynamics and physiology of saxitoxin production by the dinoflagellates *Alexandrium* spp.. Mar. Biol..

[67] Abassi S., Ki J.-S. (2022). Increased nitrate concentration differentially affects cell growth and expression of nitrate transporter and other nitrogen-related genes in the harmful dinoflagellate *Prorocentrum minimum*. Chemosphere.

[68] Prakash A.A., Rashid M. (1968). Influence of humic substances on the growth of marine phytoplankton: Dinoflagellates. Limnol. Oceanogr..

[69] Mustakim G.R., Shaleh S.R.M., Ayub M.N.A. (2019). Effect of different concentration of soil extracts on the growth of *Pyrodinium bahamense* var. Compressum. Int. J. Fish. Aquat. Sci..

[70] Gagnon R., Levasseur M., Weise A.M., Fauchot J., Campbell P.G., Weissenboeck B.J., Merzouk A., Gosselin M., Vigneault B. (2005). Growth stimulation of *Alexandrium tamarense* (*Dinophyceae*) by humic substances from the Manicouagan river (Eastern Canada). J. Phycol..

[71] Carlsson P., Edling H., Béchemin C. (1998). Interactions between a marine dinoflagellate (*Alexandrium catenella*) and a bacterial community utilizing riverine humic substances. Aquat. Microb. Ecol..

[72] Doblin M., Legrand C., Carlsson P., Hummert C., Graneli E., Hallegraeff G., Hallegraeff G.M., Blackburn S.I., Bolch C.J., Lewis R.J. (2001). Uptake of humic substances by the toxic dinoflagellate *Alexandrium catenella*. Harmful Algal Blooms 2000.

[73] Murata A., Leong S.C., Nagashima Y., Taguchi S. (2006). Nitrogen:Phosphorus supply ratio may control the protein and total toxin of dinoflagellate *Alexandrium tamarense*. Toxicon.

[74] Ou L., Wang D., Huang B., Hong H., Qi Y., Lu S. (2008). Comparative study of phosphorus strategies of three typical harmful algae in Chinese coastal waters. J. Plankton Res..

[75] Jauzein C., Labry C., Youenou A., Quéré J., Delmas D., Collos Y. (2010). Growth and phosphorus uptake by the toxic dinoflagellate Alexandrium catenella (*Dinophyceae*) in response to phosphate limitation. J. Phycol..

[76] Labry C., Erard–Le Denn E., Chapelle A., Fauchot J., Youenou A., Crassous M.-P., Le Grand J., Lorgeoux B. (2008). Competition for phosphorus between two dinoflagellates: A toxic *Alexandrium minutum* and a non-toxic *Heterocapsa triquetra*. J. Exp. Mar. Bio. Ecol..

[77] Flynn K., Franco J.M., Fernández P., Reguera B., Zapata M., Wood G., Flynn K.J. (1994). Changes in toxin content, biomass and pigments of the dinoflagellate *Alexandrium minutum* during nitrogen refeeding and growth into nitrogen or phosphorus stress. Mar. Ecol. Prog. Ser..

[78] Jean N., Perié L., Dumont E., Bertheau L., Balliau T., Caruana A.M., Amzil Z., Laabir M., Masseret E. (2022). Metal stresses modify soluble proteomes and toxin profiles in two Mediterranean strains of the distributed dinoflagellate *Alexandrium pacificum*. Sci. Total Environ..

[79] Rueler J.G., Ades D.R. (1987). The role of iron nutrition in photosynthesis and nitrogen assimilation in *Scenedesmus quadricauda* (*Chlorophyceae*). J. Phycol..

[80] He H., Chen F., Li H., Xiang W., Li Y., Jiang Y. (2010). Effect of iron on growth, biochemical composition and paralytic shellfish poisoning toxins production of *Alexandrium tamarense*. Harmful Algae.

[81] Yang I., Beszteri S., Tillmann U., Cembella A., John U. (2011). Growth-and nutrient-dependent gene expression in the toxigenic marine dinoflagellate *Alexandrium minutum*. Harmful Algae.

[82] Long M., Holland A., Planquette H., Santana D.G., Whitby H., Soudant P., Sarthou G., Hegaret H., Jolley D.F. (2019). Effects of copper on the dinoflagellate *Alexandrium minutum* and its allelochemical potency. Aquat. Toxicol..

[83] Taroncher-Oldenburg G., Kulis D.M., Anderson D.M. (1999). Coupling of saxitoxin biosynthesis to the G1 phase of the cell cycle in the dinoflagellate *Alexandrin fundyense*: Temperature and nutrient effects. Nat. Toxins.

[84] Ogata T., Ishimaru T., Kodama M. (1987). Effect of water temperature and light intensity on growth rate and toxicity change in *Protogonyaulax tamarensis*. Mar. Biol..

[85] Hamasaki K., Horie M., Tokimitsu S., Toda T., Taguchi S. (2001). Variability in toxicity of the dinoflagellate *Alexandrium tamarense* isolated from Hiroshima Bay, western Japan, as a reflection of changing environmental conditions. J. Plankton Res..

[86] Tillmann U., John U. (2002). Toxic effects of *Alexandrium* spp. on heterotrophic dinoflagellates: An allelochemical defence mechanism independent of PSP-toxin content. Mar. Ecol. Prog. Ser..

[87] Laabir M., Collos Y., Masseret E., Grzebyk D., Abadie E., Savar V., Sibat M., Amzil Z. (2013). Influence of environmental factors on the paralytic shellfish toxin content and profile of *Alexandrium catenella* (*Dinophyceae*) isolated from the Mediterranean Sea. Mar. Drugs.

[88] Navarro J., Munoz M., Contreras A. (2006). Temperature as a factor regulating growth and toxin content in the dinoflagellate *Alexandrium catenella*. Harmful Algae.

[89] Lilly E., Kulis D., Gentien P., Anderson D. (2002). Paralytic shellfish poisoning toxins in France linked to a human-introduced strain of *Alexandrium catenella* from the western Pacific: Evidence from DNA and toxin analysis. J. Plankton Res..

[90] Abdulhussain A.H., Cook K.B., Turner A.D., Lewis A.M., Elsafi M.A., Mayor D.J. (2020). The influence of the toxin producing dinoflagellate, *Alexandrium catenella* (1119/27), on the feeding and survival of the marine Copepod, *Acartia tonsa*. Harmful Algae.

[91] Etheridge S.M., Roesler C.S. (2005). Effects of temperature, irradiance, and salinity on photosynthesis, growth rates, total toxicity, and toxin composition for *Alexandrium fundyense* isolates from the Gulf of Maine and Bay of Fundy. Deep Sea Res. Part II Top. Stud. Oceanogr..

[92] Lim P.T., Leaw C.P., Usup G., Kobiyama A., Koike K., Ogata T. (2006). Effects of light and temperature on growth, nitrate uptake, and toxin production of two tropical dinoflagellates: *Alexandrium tamiyavanichii* and *Alexandrium minuyum* (*Dinophyceae*). J. Phycol..

[93] Hadjadji I., Laabir M., Frihi H., Collos Y., Shao Z.J., Berrebi P., Abadie E., Amzil Z., Chomérat N., Rolland J.L. (2020). Unsuspected intraspecific variability in the toxin production, growth and morphology of the dinoflagellate *Alexandrium pacificum* RW Litaker (Group IV) blooming in a South Western Mediterranean marine ecosystem, Annaba Bay (Algeria). Toxicon.

[94] Caruana A.M., Le Gac M., Hervé F., Rovillon G.-A., Geffroy S., Malo F., Abadie E., Amzil Z. (2020). *Alexandrium pacificum* and *Alexandrium minutum*: Harmful or environmentally friendly?. Mar. Environ. Res..

[95] Alkawri A., Ramaiah N. (2010). Spatio-temporal variability of dinoflagellate assemblages in different salinity regimes in the west coast of India. Harmful Algae.

[96] Errera R.M., Campbell L. (2011). Osmotic stress triggers toxin production by the dinoflagellate *Karenia brevis*. Proc. Natl. Acad. Sci. USA.

[97] Kirst G. (1990). Salinity tolerance of eukaryotic marine algae. Annu. Rev. Plant Biol..

[98] Lim P.T., Leaw C.P., Sato S., Thuoc C.V., Kobiyama A., Ogata T. (2011). Effect of salinity on growth and toxin production of *Alexandrium minutum* isolated from a shrimp culture pond in northern Vietnam. J. Appl. Phycol..

[99] Lim P.-T., Ogata T. (2005). Salinity effect on growth and toxin production of four tropical *Alexandrium* species (Dinophyceae). Toxicon.

[100] Shin H.H., Baek S.H., Zhun L., Han M.-S., Oh S.J., Youn S.-H., Kim Y.S., Kim D., Lim W.-A. (2014). Resting cysts, and effects of temperature and salinity on the growth of vegetative cells of the potentially harmful species *Alexandrium insuetum* Balech (Dinophyceae). Harmful Algae.

[101] Glaser K., Karsten U. (2020). Salinity tolerance in biogeographically different strains of the marine benthic *diatom Cylindrotheca closterium* (Bacillariophyceae). J. Appl. Phycol..

[102] Aguilera-Belmonte A., Inostroza I., Carrillo K.S., Franco J.M., Riobó P., Gómez P.I. (2013). The combined effect of salinity and temperature on the growth and toxin content of four Chilean strains of *Alexandrium catenella* (Whedon and Kofoid) Balech 1985 (Dinophyceae) isolated from an outbreak occurring in southern Chile in 2009. Harmful Algae.

[103] Grzebyk D., Béchemin C., Ward C.J., Vérité C., Codd G.A., Maestrini S.Y. (2003). Effects of salinity and two coastal waters on the growth and toxin content of the dinoflagellate *Alexandrium minutum*. J. Plankton Res..

[104] Hwang D.F., Lu Y.H. (2000). Influence of environmental and nutritional factors on growth, toxicity, and toxin profile of dinoflagellate *Alexandrium minutum*. Toxicon.

[105] Lazcano-Ferrat I., Lovatt C.J. (1997). Effect of salinity on Arginine biosynthesis in leaves of *Phaseolus vulgaris* LP acutifolius A. Gray. Crop Sci..

[106] Martens H., Van de Waal D.B., Brandenburg K.M., Krock B., Tillmann U. (2016). Salinity effects on growth and toxin production in an *Alexandrium ostenfeldii* (Dinophyceae) isolate from The Netherlands. J. Plankton Res..

[107] Murray S.A., Diwan R., Orr R.J., Kohli G.S., John U. (2015). Gene duplication, loss and selection in the evolution of saxitoxin biosynthesis in alveolates. Mol. Phylogenet. Evol..

[108] Wiese M. (2012). Investigations into Abiotic and Biotic Factors Regulating Saxitoxin Synthesis in the Dinoflagellate Genus *Alexandrium*. Ph.D. Thesis.

[109] Glover H., Beardall J., Morris I. (1975). Effects of environmental factors on photosynthesis patterns in *Phaeodactylum tricornutum* (bacillariophyceae). I. Effect of nitrogen deficiency and light intensity. J. Phycol..

[110] Vingiani G.M., Štālberga D., De Luca P., Ianora A., De Luca D., Lauritano C. (2020). De novo transcriptome of the non-saxitoxin producing *Alexandrium tamutum* reveals new insights on harmful dinoflagellates. Mar. Drugs.

[111] Akbar M.A., Yusof N.Y.M., Sahrani F.K., Usup G., Ahmad A., Baharum S.N., Muhammad N.A.N., Bunawan H. (2021). Insights into *Alexandrium minutum* nutrient acquisition, metabolism and saxitoxin biosynthesis through comprehensive transcriptome survey. Biology.

[112] Oh S.J., Kim D.-I., Sajima T., Shimasaki Y., Matsuyama Y., Oshima Y., Honjo T., Yang H.-S. (2008). Effects of irradiance of various wavelengths from light-emitting diodes on the growth of the harmful dinoflagellate *Heterocapsa circularisquama* and the diatom *Skeletonema costatum*. Fish. Sci..

[113] McGillicuddy D.J., Anderson D.M., Lynch D.R., Townsend D.W. (2005). Mechanisms regulating large-scale seasonal fluctuations in *Alexandrium fundyense* populations in the Gulf of Maine: Results from a physical–biological model. Deep Sea Res. Part II Top. Stud. Oceanogr..

[114] Prézelin B.B., Alberte R.S. (1978). Photosynthetic characteristics and organization of chlorophyll in marine dinoflagellates. Proc. Natl. Acad. Sci. USA.

[115] Prézelin B.B. (1976). The role of peridinin-chlorophyll a-proteins in the photosynthetic light adaption of the marine dinoflagellate, *Glenodinium* sp.. Planta.

[116] Rivkin R.B. (1989). Influence of irradiance and spectral quality on the carbon metabolism of phytoplankton. I. Photosynthesis, chemical composition and growth. Mar. Ecol. Prog. Ser. Oldendorf.

[117] Burja A.M., Banaigs B., Abou-Mansour E., Burgess J.G., Wright P.C. (2001). Marine cyanobacteria—A prolific source of natural products. Tetrahedron.

[118] Salgado P., Vázquez J.A., Riobó P., Franco J.M., Figueroa R.I., Kremp A., Bravo I. (2015). A kinetic and factorial approach to study the effects of temperature and salinity on growth and toxin production by the dinoflagellate *Alexandrium ostenfeldii* from the Baltic Sea. PLoS ONE.

[119] Nam K.T., Kim S.-Y., Moon C.-H., Kim C.-H., Oh S.J. (2020). Effects of light wavelengths on the growth and paralytic shellfish toxin production of *Alexandrium catenella* and *A. pacificum*. J. Korean Soc. Mar. Environ..

[120] Pistocchi R., Rossini G.P. (2014). Factors affecting algal toxicity. Toxins and Biologically Active Compound from Microalgae.

[121] Alpermann T.J., Tillmann U., Beszteri B., Cembella A.D., John U. (2010). Phenotypic variation and genotypic diversity in a planktonic population of the toxigenic marine dinoflagellate *Alexandrium tamarense* (Dinophyceae). J. Phycol..

[122] Gao Y., Sassenhagen I., Richlen M.L., Anderson D.M., Martin J.L., Erdner D.L. (2019). Spatiotemporal genetic structure of regional-scale *Alexandrium catenella* dinoflagellate blooms explained by extensive dispersal and environmental selection. Harmful Algae.

[123] Kremp A., Oja J., LeTortorec A.H., Hakanen P., Tahvanainen P., Tuimala J., Suikkanen S. (2016). Diverse seed banks favour adaptation of microalgal populations to future climate conditions. Environ. Microbiol..

[124] Iglesias-Prieto R., Trench R. (1997). Acclimation and adaptation to irradiance in symbiotic dinoflagellates. II. Response of chlorophyll–protein complexes to different photon-flux densities. Mar. Biol..

[125] Kellmann R., Mihali T.K., Jeon Y.J., Pickford R., Pomati F., Neilan B.A. (2008). Biosynthetic intermediate analysis and functional homology reveal a saxitoxin gene cluster in cyanobacteria. Appl. Environ. Microbiol..

[126] Kellmann R., Michali T.K., Neilan B.A. (2008). Identification of a saxitoxin biosynthesis gene with a history of frequent horizontal gene transfers. J. Mol. Evol..

[127] Schantz E.J., Mold J.D., Stanger D.W., Shavel J., Riel F.J., Bowden J.P., Lynch J.M., Wyler R.S., Riegel B., Sommer H. (1957). Paralytic shellfish poison. VI. A procedure for the isolation and purification of the poison from toxic clam and mussel tissues. J. Am. Chem. Soc..

[128] Moustafa A., Loram J.E., Hackett J.D., Anderson D.M., Plumley F.G., Bhattacharya D. (2009). Origin of saxitoxin biosynthetic genes in cyanobacteria. PLoS ONE.

[129] Hackett J.D., Wisecaver J.H., Brosnahan M.L., Kulis D.M., Anderson D.M., Bhattacharya D., Plumley F.G., Erdner D.L. (2013). Evolution of saxitoxin synthesis in cyanobacteria and dinoflagellates. Mol. Biol. Evol..

[130] Zhang Y., Zhang S.-F., Lin L., Wang D.-Z. (2014). Comparative transcriptome analysis of a toxin-producing dinoflagellate *Alexandrium catenella* and its non-toxic mutant. Mar. Drugs.

[131] Zhang Y., Zhang S.-F., Lin L., Wang D.-Z. (2017). Whole transcriptomic analysis provides insights into molecular mechanisms for toxin biosynthesis in a toxic dinoflagellate *Alexandrium catenella* (ACHK-T). Toxins.

[132] Wang D.-Z., Zhang S.-F., Zhang Y., Lin L. (2016). Paralytic shellfish toxin biosynthesis in cyanobacteria and dinoflagellates: A molecular overview. J. Proteom..

[133] Verma A., Barua A., Ruvindy R., Savela H., Ajani P.A., Murray S.A. (2019). The genetic basis of toxin biosynthesis in dinoflagellates. Microorganisms.

[134] Kim H.S., Park H., Wang H., Kim T., Ki J.S. (2023). Saxitoxins-producing potential of the marine dinoflagellate *Alexandrium affine* and its environmental implications revealed by toxins and transcriptome profiling. Mar. Environ. Res..

[135] Bui Q.T.N., Kim H., Wang H., Ki J.S. (2022). Unveiling the genomic structures and evolutionary events of the saxitoxin biosynthetic gene sxtA in the marine toxic dinoflagellate Alexandrium. Mol. Phylogenet. Evol..

[136] Kim H.S., Bui Q.T.N., Wang H., Ki J.S. (2023). Molecular cloning, origin, and expression of saxitoxin biosynthesis gene *sxtB* from the toxic dinoflagellate *Alexandrium catenella*. J. Appl. Phycol..

[137] Mihali T.K., Kellmann R., Neilan B.A. (2009). Characterisation of the paralytic shellfish toxin biosynthesis gene clusters in *Anabaena circinalis* AWQC131C and *Aphanizomenon* sp. NH-5. BMC Biochem..

[138] Mihali T.K., Carmichael W.W., Neilan B.A. (2011). A putative gene cluster from a *Lyngbya wollei* bloom that encodes paralytic shellfish toxin biosynthesis. PLoS ONE.

[139] Cullen A., D’Agostino P.M., Mazmouz R., Pickford R., Wood S., Neilan B.A. (2018). Insertions within the saxitoxin biosynthetic gene cluster result in differential toxin profiles. ACS Chem. Biol..

[140] Chekan J.R., Fallon T.R., Moore B.S. (2020). Biosynthesis of marine toxins. Curr. Opin. Chem. Biol..

[141] Orr R.J., Stüken A., Murray S.A., Jakobsen K.S. (2013). Evolutionary acquisition and loss of saxitoxin biosynthesis in dinoflagellates: The second “core” gene, *sxtG*. Appl. Environ. Microbiol..

[142] Akbar M.A., Mohd Yusof N.Y., Tahir N.I., Ahmad A., Usup G., Sahrani F.K., Bunawan H. (2020). Biosynthesis of saxitoxin in marine dinoflagellates: An omics perspective. Mar. Drugs.

[143] Kim H.S., Bui Q.T.N., Shin J., Wang H., Ki J.S. (2024). Promoter regions of *sxtA* and *sxtG* reveal relationship between saxitoxin biosynthesis and photosynthesis in toxic *Alexandrium catenella*. J. Appl. Phycol..

[144] Wang H., Kim H., Ki J.-S. (2021). Preliminary result of de novo transcriptome sequencing of the marine toxic dinoflagellate *Alexandrium catenella* incubated under several different stresses. Mar. Biol..

[145] Naves J.L., Prado M.P., Rangel M., De Sanctis B., Machado-Santelli G., Freitas J.C. (2006). Cytotoxicity in the marine dinoflagellate *Prorocentrum mexicanum* from Brazil. Comp. Biochem. Physiol. C Toxicol. Pharmacol..

[146] Bui Q.T.N., Ki J.S. (2023). Molecular characterization and expression analysis of saxitoxin biosynthesis gene *sxtU* from toxigenic dinoflagellate *Alexandrium pacificum*. J. Appl. Phycol..

